# Purulent and Gonorrhœal Conjunctivitis

**Published:** 1873-04

**Authors:** 


### SELECTIONS.

### PURULENT AND GONORRHŒAL CONJUNCTIVITIS.

BY PROFESSOR SEELY.

*A Clinical Lecture at the Dispensary of the Medical College of Ohio.*

I am unable to tell you, gentlemen, why, as the result of a given cause, we find in one case one form of inflammation, and in another case some other form.

I present you to-day with two cases of inflammation of the conjunctiva, attended with a purulent discharge.

The first case, as you see, presents all the symptoms of a violent inflammation, viz., great swelling of the lids, which are hot and œdematous, the eyelashes matted together, great chemosis of the bulbous conjunctiva, and an abundant discharge.

On questioning the patient, we make out no infection by gonorrhœal matter, but find that he has had weak eyes for some time, and that, being very much exposed, the eyes suddenly became greatly swollen, and in a day or two began to discharge freely.

While speaking of granulations, I told you that a possible termination to such a condition might in the appearance of all the symptoms be presented here. I also remarked that the manner in which granulations appeared was through absorption, and hence a certain amount of inflammation was necessary to rid the conjunctiva of the new growths. I also spoke of the process of getting rid of these bodies—the so-called inoculation method. I have also shown you how moist warmth may be applied to bring about a similar state of things.

If my diagnosis is correct, we have here a purulent inflammatory process engrafted upon the trachoma. Everything seems to point in that direction. When we evert the lids, we are unable to *see* any granulations, it is true, because the papillæ are considerably enlarged; and yet, when you have seen a few cases of trachomatous conjunctiva converted into the purulent, and of the papillated the result of purulent primary inflammation, you will be able to see that something which I think indicates a difference.

Here, as is usual, we have the statement that the eyes had been weak for some time.

While speaking of this possible termination of granulations, I specially called your attention to the points in the therapeutical management, for in this resides the chief interest.

In these conjunctival inflammations, the danger is in complications on the part of the cornea.

Let us look, then, for a few moments, at the points in treatment. In this country, we see but seldom that terrible inflammation that attacks the conjunctiva, the diphtheritic, characterized by extreme hardness of the lid, due to a fibrinous exudation into the conjunctiva and sub-conjunctival tissue, bulbous as well as palpebral, that may bring about destruction of the cornea, in some cases, in thirty-six hours.

However, we not unfrequently do see symptoms which indicate that we are in the presence of more or less stasis in the circulation.

In the stage of inflammation and swelling that immediately precedes the purulent, in purulent conjunctivitis then, the point is to beware of the use of astringents; in fact, I may say that is the safe general rule that cannot be insisted upon too strongly. In this stage your reliance must be upon a general local antiphlogistic treatment. A brisk purgative or two, composed in part of calomel. Opium for the relief of pain.

Quiet in bed, with the room moderately darkened.

Locally, if the patient is in a physical condition that justifies it, blood-letting by leeches or cups. If the lids are greatly swollen, a lengthening of the commissure by slitting, which of itself may let out the necessary amount of blood.

As a wash, the sulphate of atropia. (gr. jv. ad. ℥j).

I say nothing of the application of cold, as it requires such great care to be followed by beneficial results.

After the purulent stage begins, indicated by the appearance of pus, your more active local interference comes in.

The same cautioning at the beginning of this stage applies in this country as well as in Europe; "begin your astringents carefully," for fear of producing a stasis in the circulation and changing the resolution stage into a violently active one, that gives rise to layers of lymph upon the surface of the conjunctiva, and possibly a fibrinous exudation into its substance.

As to the therapeutic agents, the one generally recommended in all purulent inflammations is nitrate of silver. Then begin by applying a weak solution of the nitrate (gr. v. ad. ℥j.), on the appearance of the purulent discharge, brushing it upon the everted lids, or on the lower if they cannot be everted on account of the swelling.

If the next day the purulency has increased, you can apply a stronger solution, and neutralize it with salt and water. When the symptoms of acuteness have began after a few days to subside, your antiphlogistic treatment should be abandoned.

For some little time after your brushing with the nitrate, you can apply cold compresses to the lids, changing them every minute or two. The atropia and brushings with the nitrate should be continued until the purulent discharge has entirely ceased.

If complications on the part of the cornea arise, there will be no reason for a discontinuance of the brushings, only more care must be taken not to make pressure on the cornea, and not to allow any of the nitrate to touch it. If there is an ulcer forming, the lower lid can still be brushed, and a compress bandage applied firmly over the eye, to be removed several times a day to cleanse the eye.

This will probably be all that most of you will be able to do; of course, by such treatment you will only have met one part of the indications, for there is needed not only support to the weakened tissue to help it to bear the intra-ocular tension, but also a reduction of this intra-ocular tension, which is to some extent accomplished by the atropia.

So, if it is possible, a paracentesis of the anterior chamber should be made.

If the ulcer is small, a perforation may take place beneath the bandage and no harm result further than a small opacity.

If the ulcer has perforated, and there is a prolapse of the iris, it is best to puncture it, then snip it off with the scissors.

What shall be our course in the case before us as regards the therapeutic management? I am specially glad of an opportunity to illustrate the *differential* treatment, if you will allow me the expression, between such a case and one of idiopathic purulent conjunctivitis. If my diagnosis is correct, we have a state of things that will rid the patient of these granulations, provided we treat him properly. What is indicated? What are we to do? I take it we are simply to be on guard to prevent mischief.

We will prescribe a solution of atropine (gr. vjj. ad. ℥j.), and simply have the eye kept clean.

If we cut short the suppurative stage, we will interfere with the production of the very result hoped for, viz., the absorption of the granulations.

Then in such cases keep in view what is to be accomplished, and only interfere when danger threatens—for example, implicatiom of the cornea.

Gonorrhœal Ophthalmia.

Here is an illustrative case. Not very violent, for, fortunately, the patient's eyes have withstood the worst of the siege. We still have quite an abundant discharge of pus, but the lids are not nearly so swollen as they were two days ago.

This disease is undoubtedly one of the gravest that attacks the eye. It is, if you please, an exaggerated purulent conjunctivitis. The cause is very virulent, and hence we have a correspondingly active inflammation.

The early symptoms differ little from those usually given for other inflammations of the conjunctiva, but pass on far more rapidly to the characters that correspond to so grave a disease, viz., extreme swelling of the lids; they are hot, red and œdematous; the conjunctiva is swollen and greatly chemosed, so that the cornea is almost hidden by its rolls.

The purulent discharge is thick and yellow, profuse, running out upon the cheeks as it does in this patient's right eye when the lids are opened.

You see that we are really in the presence of symptoms of a violent purulent conjunctivitis. The history of the case makes us call it gonorrhœal ophthalmia. An inflammation produced by getting some of the urethral pus into the eye.

Gonorrhœal ophthalmia is a purulent conjunctivitis produced by contagion in the manner just stated.

Even the discharge from the urethra termed gleet, the remains of gonorrhœa, may produce a violent purulent conjunctivitis. So patients should always be on their guard so long as any discharge remains.

We have seen, then, that here we have to do with an exaggerated purulent conjunctivitis, differing in degree, but not in kind. What are the therapeutic indications? If symptoms of conjunctival irritation appear in a patient suffering from gonorrhœa, they must be immediately combatted by morphia or atropia solutions; if considerable swelling is already present, I would recommend the same kind of treatment that I have given for commencing ophthalmia neonatorum, a weak solution of nitrate of silver used several times a day; even brushing the lids with a moderately strong solution, and afterwards using ice or ice-cold compresses.

The further indications differ in no respect from those in purulent conjunctivitis.—*Clinic.*

